# One-year Mortality Outcomes From the Advancing Cryptococcal Meningitis Treatment for Africa Trial of Cryptococcal Meningitis Treatment in Malawi

**DOI:** 10.1093/cid/ciz454

**Published:** 2019-06-01

**Authors:** Cecilia Kanyama, Síle F Molloy, Adrienne K Chan, Duncan Lupiya, Chimwemwe Chawinga, Jack Adams, Philip Bright, David G Lalloo, Robert S Heyderman, Olivier Lortholary, Shabbar Jaffar, Angela Loyse, Joep J van Oosterhout, Mina C Hosseinipour, Thomas S Harrison

**Affiliations:** 1 University of North Carolina Project-Malawi, Kamuzu Central Hospital, Lilongwe; 2 Centre for Global Health, Institute for Infection and Immunity, St George’s University of London, United Kingdom; 3 Dignitas International, Zomba Central Hospital, Malawi; 4 Division of Infectious Diseases, Department of Medicine, Sunnybrook Health Sciences Centre, University of Toronto, Canada; 5 Clinical Immunology Department, North Bristol National Health Service Trust, United Kingdom; 6 Liverpool School of Tropical Medicine, United Kingdom; 7 Malawi-Liverpool-Wellcome Trust Clinical Research Programme, Blantyre; 8 Department of Medicine, College of Medicine, University of Malawi, Blantyre; 9 University College London, United Kingdom; 10 Institut Pasteur, Molecular Mycology Unit, National Reference Center for Invasive Mycoses & Antifungals, Centre National de la Recherche Scientifique, Paris; 11 Paris Descartes University, Necker Pasteur Center for Infectious Diseases and Tropical Medicine, Institut Hospitalo-Universitaire Imagine, Assistance Publique - Hôpitaux de Paris, France; 12 Division of Infectious Diseases, School of Medicine, University of North Carolina at Chapel Hill

**Keywords:** cryptococcal meningitis, treatment, long-term follow-up, Malawi, HIV

## Abstract

In Malawi, 236 participants from the Advancing Cryptococcal Meningitis Treatment for Africa trial were followed for 12 months. The trial outcomes reported at 10 weeks were sustained to 1 year. One-week amphotericin B plus flucytosine was associated with the lowest 1 year mortality (27.5% [95% confidence interval, 16.3 to 44.1]).

Mortality from cryptococcal meningitis (CM) remains unacceptably high in sub-Saharan Africa [1]. The most widely used treatment, fluconazole (FLU) monotherapy, is associated with mortality of 50%–60% at 10 weeks and >70% at 1 year [2–5]. The Advancing Cryptococcal Meningitis Treatment for Africa (ACTA) trial [6] recently tested new induction treatment strategies against the 2010 recommended standard of 2 weeks amphotericin B (AmB)–based induction treatment [7]. The new treatment strategies were FLU plus flucytosine (5FC) given orally for 2 weeks and AmB given for 1 week with either FLU or 5FC. The aim was to improve outcomes in CM with regimens that could be sustained in resource-limited settings. At 10 weeks of follow-up, results from the trial showed that the oral combination was noninferior to 2 weeks AmB+ 5FC. One week of AmB+5FC was associated with the lowest mortality overall. While 10-week mortality is the most commonly used endpoint in CM trials, there is less controlled data on longer term outcomes. Here, we report the results at 12 months of follow-up for participants enrolled at 2 centers in Malawi, comprising a subset of all those enrolled in the trial.

## METHODS

ACTA was a large, open-label, phase 3, randomized, noninferiority, multicenter trial in which 721 patients with human immunodeficiency virus (HIV)–associated CM from centers in Malawi, Zambia, Tanzania, and Cameroon were randomized to 3 strategies: oral FLU+5FC for 2 weeks, 1 week AmB, and standard 2 weeks AmB. Those in the AmB arms were further randomized to 5FC or FLU in a 1:1 ratio, as the partner drug given with AmB. Outcomes at 10 weeks of antifungal therapy have been reported [6]. In this preplanned substudy of the ACTA trial, participants at 2 centers in Malawi (Kamuzu Central Hospital, Lilongwe and Zomba Central Hospital where there were sufficient resources for appropriate and complete follow-up), representing almost one-third of patients from the main trial were followed up for 12 months, with primary endpoints of all-cause mortality at 6 and 12 months.

### Data Collection

Trained study personnel (nurse or clinician) collected data using a structured questionnaire and record review. Face-to-face interviews were conducted at 6 and 12 months posttrial entry. If face-to-face interviews were not possible, patients were followed up by telephone (see Supplementary Materials for details).

### Statistical Analyses

Stata 14.2 (StataCorp LP, College Station, TX) was used for statistical analyses. All-cause mortality at 10 weeks, 6 months, and 1 year was compared between the treatment groups using log-rank tests. Kaplan-Meier plots were constructed, and Cox regression models with treatment as a predictor were used to derive hazard ratios (HRs) and 95% confidence intervals (CIs). The analyses were also adjusted for the following prespecified baseline covariates: site, age, sex, Glasgow coma scale, CD4+ cell count, cerebrospinal fluid fungal count, and antiretroviral therapy (ART) status. Analyses were performed on both intention-to-treat (ITT) and per-protocol populations, as in the main trial analyses [6]. Analyses were undertaken to explore the consistency of the main trial results over time.

### Ethics

The London School of Hygiene and Tropical Medicine Research Ethics Committee and the national ethics and regulatory bodies in each country approved the ACTA trial protocol [6]. Ethical approvals for this substudy were obtained from the National Health Sciences Research Committee of Malawi and the University of North Carolina School of Medicine Chapel Hill Institutional Review Board. Written informed consent was obtained from all participants or their closest living relative.

## RESULTS

From January 2013 to November 2016, 236 patients were randomized, 186 patients in Lilongwe and 50 in Zomba (Supplementary Figure 1). Of these, 12 patients were excluded from all analyses, as in the main trial [6]; 4 did not meet inclusion criteria (2 had previous CM and 2 were CM negative), 7 met predefined late exclusion criteria, and 1 immediately withdrew consent. Thus, 224 patients formed the ITT population. In total, 144 patients were alive at 10 weeks and all were followed up at 1 year, with no losses to follow-up.

Baseline characteristics were consistent with those of the trial as a whole (Supplementary Tables 1, 3, and 5). In total, 63% (141/224) of patients reported that they were taking, or had previously taken, ART. Patients who said that they had never taken ART started ART at a median of 28 days (interquartile range, 27–32) after randomization. Of the 123 participants who survived to 1 year, ART adherence data were available for 113; 109 participants (96.5%) were reportedly fully adherent. Of the 21 participants who died between 10 weeks and 1 year, 61.9% (13/21) were reportedly fully adherent, with no obvious imbalance of nonadherent participants between the arms.

**Figure 1.**
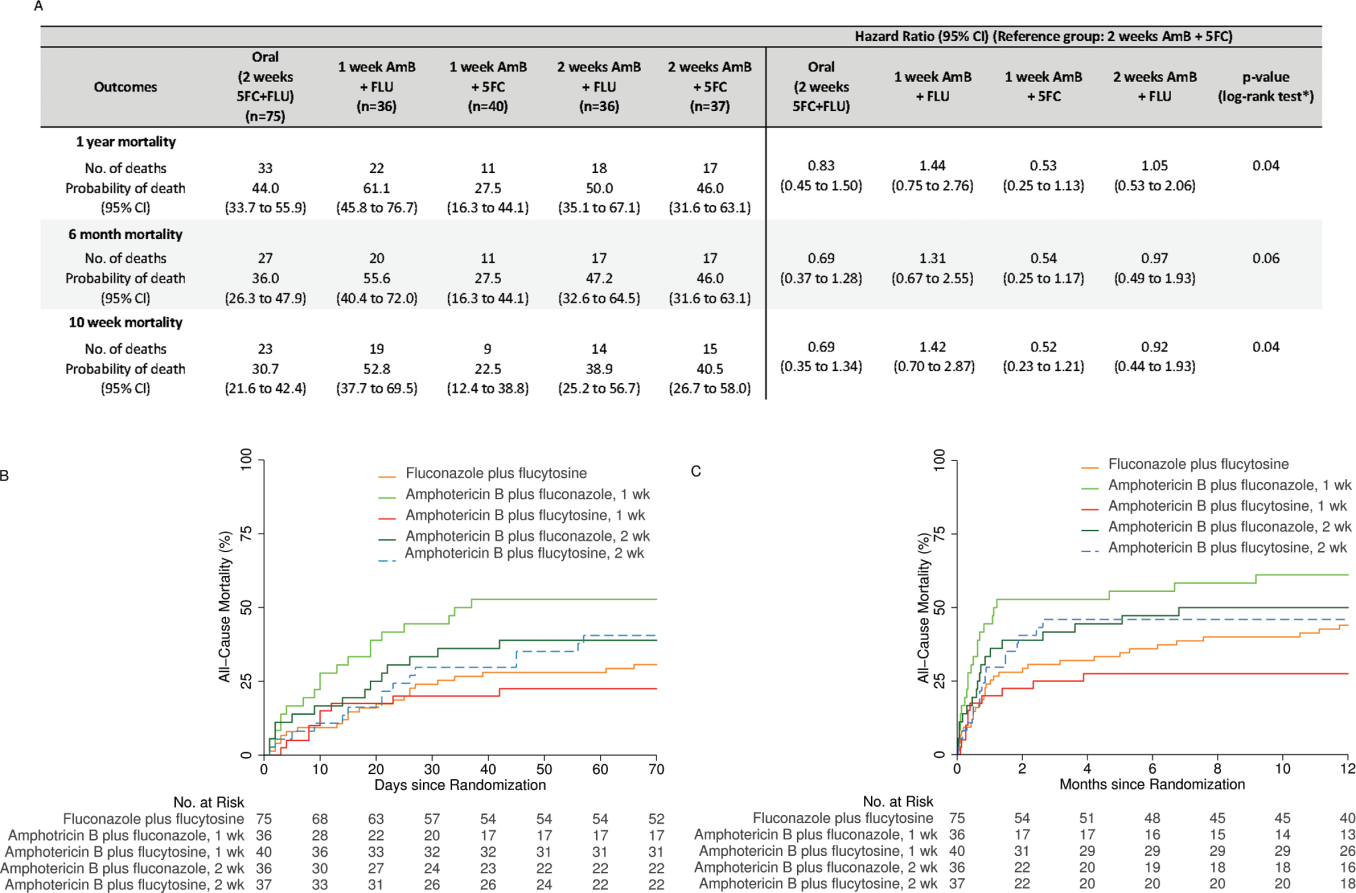
(*A*) Time to event outcomes by 5 treatment arms (intention-to-treat, adjusted analysis; N = 224). The cumulative all-cause mortality by 5 treatment arms up to 10 weeks (*B*) and 1 year (*C*) post-randomization. Abbreviations: 5FC, flucytosine; AmB, amphotericin B; CI, confidence interval; FLU, fluconazole. *Log rank *P* value for the unadjusted analysis.

Mortality at 10 weeks followed the main trial results. Supplementary Tables 4 and 6 show results for the comparison of the 3 treatment strategies and for the comparison of the partner drug, FLU or 5FC, given with AmB. Figure 1A and B and Supplementary Table 2 show the results for the comparison between the 5 treatment arms.

Overall mortality was 35.7% at 10 weeks (95% CI, 29.4 to 42.4; n = 80/224), 41.1% at 6 months (95% CI, 35.0 to 47.8; n = 92/224), and 45.1% at 1 year (95% CI, 38.9 to 51.8; n = 101/224). Thus, of those who survived to 10 weeks, 85% (123/144) survived to 1 year. Results at 10 weeks were sustained to 6 and 12 months. In particular, results for the 5 treatment arms are shown in Figure 1A (adjusted analyses with 2 weeks AmB+5FC as the comparator; results with 1 week AmB+5FC as the comparator are shown in Supplementary Table 2) and Figure 1B and C. One week AmB+5FC was associated with a 12-month mortality of 27.5% (95% CI, 16.3 to 44.1), which was not statistically significantly different from that in the other arms. HRs for mortality, comparing 2 weeks AmB+5FC with other treatment arms, were similar at 6 and 12 months to those at 10 weeks; for example, comparing 2 weeks AmB+5FC with 1 week AmB+5FC, HRs (95% CI) were 1.91 (0.83 to 4.39), 1.84 (0.86 to 3.95), and 1.90 (0.88 to 4.08) at 10 weeks, 6 months, and 12 months, respectively (Supplementary Table 2).

These HRs are similar to those for this comparison in the trial as a whole at 10 weeks (HR, 1.79; 95% CI, 1.10 to 2.89 [6]). Results at all time points were similar for adjusted and unadjusted and for ITT and per-protocol analyses. When mortality between 10 weeks and 1 year was analyzed, there was no difference between groups, as expected, although 1 week AmB+5FC had the lowest proportion of late deaths (2/31, 6%).

Comparing AmB partner drugs, the hazard of death was significantly lower for participants administered 5FC compared to those taking FLU at all time points (1 year: HR, 0.56; 95% CI, 0.34 to 0.91; log-rank *P* value, 0.02; Supplementary Table 6).

There was no evidence for a difference in mortality at 12 months between participants who had previously been exposed to ART (43.4%) and unexposed participants (48.2%; HR, 0.89; 95% CI, 0.60 to 1.33; log rank *P* value, 0.58; Supplementary Figure 2), though numbers are low.

## DISCUSSION

Drug treatment effects observed at 10 weeks were sustained at 12 months, with remarkably low mortality (27.5%) evident in the 1-week AmB+5FC arm. This substudy, with complete follow-up of 224 participants to 1 year post-induction therapy, extends the results seen at 10 weeks in the ACTA trial overall. The long-term benefit seen with 1 week AmB+5FC supports updated World Health Organization (WHO) guidelines that recommend this regimen as first-line induction therapy and underlines the need for rapid wide access to 5FC [8, 9].

The results also confirm that after 10 weeks, mortality rates decrease, with a general flattening out of survival curves in the context of effective antifungal therapy and appropriately timed ART (ie, 4 weeks after the start of antifungal therapy). The few long-term follow-up data that are available in the context of timely ART initiation for all patients are consistent with our study in this regard [10, 11]. The long-term mortality curves in all the regimens tested here contrast with those from studies that used FLU monotherapy, in which progressively increasing mortality resulted in mortality at 12 months of more than 75% [4], further emphasizing the benefit of currently recommended regimens over FLU monotherapy. Given the relatively low mortality between 10 weeks and 1 year, our results also suggest that life-threatening CM-immune reconstitution inflammatory syndrome reactions are uncommon or can usually be managed with awareness and appropriate investigation and treatment [12].

No differences in long-term survival were detected between participants previously exposed to ART and ART-unexposed participants. Further work is needed to separate out, within the ART exposed group, those with unmasking CM-IRIS and those who failed ART due to adherence or resistance issues. Our results underline that all these patients can have a good long-term prognosis if they survive meningitis. Thus, continued efforts are needed to target and support patients with advanced HIV disease, as recently emphasized by WHO and others [8, 9]. Last, significantly lower mortality was observed for participants administered 5FC compared to those taking FLU as a partner drug with AmB at all time points, underlining the urgent need to make 5FC readily available for patients with CM.

## Supplementary Data

Supplementary materials are available at *Clinical Infectious Diseases* online. Consisting of data provided by the authors to benefit the reader, the posted materials are not copyedited and are the sole responsibility of the authors, so questions or comments should be addressed to the corresponding author.

ciz454_suppl_Supplementary_Appendix

## Notes

***Acknowledgments.*** The Advancing Cryptococcal Meningitis Treatment for Africa trial was supported by grants from the Medical Research Council, United Kingdom (100504) and the French Agency for Research on AIDS and Viral Hepatitis (ANRS) (ANRS12275). The authors thank all the patients and their families; the nursing team of Emily Kumwenda, Grace Kaphale, and Eliana Nyondo; Andrew Nunn, Halima Dawood, Andrew Kitua, and William Powderly for serving on the data and safety monitoring committee; and Graeme Meintjes, Calice Talom, Newton Kumwenda, and Maryline Bonnet for serving on the trial steering committee.

***Potential conflicts of interest.*** T. S. H. has received speaker fees from Pfizer and Gilead, consultancy fees from Viamet, and a grant from Gilead outside the submitted work. All other authors report no potential conflicts. The authors have submitted the ICMJE Form for Disclosure of Potential Conflicts of Interest. Conflicts that the editors consider relevant to the content of the manuscript have been disclosed.
