# Supplementary material for: One-year Mortality Outcomes From the Advancing Cryptococcal Meningitis Treatment for Africa Trial of Cryptococcal Meningitis Treatment in Malawi
Source: Clin Infect Dis. 2019 Jun 1;70(3):521–4. doi: 10.1093/cid/ciz454 (PMC7105249; doi:10.1093/cid/ciz454)
Supplement: ciz454_suppl_Supplementary_Appendix [file ciz454_suppl_supplementary_appendix.docx]

**SUPPLEMENTARY APPENDIX**

**TABLE OF CONTENTS**

[Data Collection 2](#_Toc8038712)

[Figure 1. Screening, Randomization, and Analysis Populations 3](#_Toc8038713)

[Table 1. Baseline characteristics of study participants by 5 treatment arms (N=224) 4](#_Toc8038714)

[Table 2. Time to event outcomes by 5 treatment arms (Intention-to-treat, adjusted analysis, 1 week AmB+5FC as reference; N=224) 5](#_Toc8038715)

[Table 3. Baseline characteristics of study participants by 3 treatment regimens (N=224) 6](#_Toc8038716)

[Table 4. Time to event outcomes by 3 treatment regimens (Intention-to-treat, unadjusted analysis; N=224) 7](#_Toc8038717)

[Table 5. Baseline characteristics of study participants by AmB partner treatment (N=149) 8](#_Toc8038718)

[Table 6. Time to event outcomes by AmB partner treatment (Intention-to-treat, unadjusted analysis; N=149) 9](#_Toc8038719)

[Figure 2. All-Cause Mortality by ARV Exposure 9](#_Toc8038720)

# Data Collection

Trained study personnel (nurse or clinician) collected data using a structured questionnaire and record review. Face-to-face interviews were conducted at 6 and 12 months post-trial entry. If face-to-face interviews were not possible patients were followed up by telephone. The vital status of participants, access to antiretroviral therapy (ART) and fluconazole, baseline and follow up CD4 cell counts and HIV viral load results, when available, were ascertained. Data were double entered into a Microsoft Access database 2016 (Microsoft, Redmond, WA, USA) and predetermined queries for validation were used.

334 Patients were assessed for eligibility

98 were excluded

6 Were <18yrs

24 previous CM

6 unwilling consent HIV test

26 declined to participate

1 on ART

3 pregnant or lactating

2 received AmB in previous 2 weeks

37 received FLU in previous 2 weeks

75 Were included in the intention-to-treat analysis

73 Were included in the intention-to-treat analysis

36 Received amphotericin B plus fluconazole

37 Received amphotericin B plus flucytosine

79 Were assigned to receive oral fluconazole plus flucytosine for 2 weeks

75 Were eligible for the trial

4 Were excluded

1 Did not have confirmed cryptococcal meningitis

2 Met late-exclusion criteria

1 Had previous cryptococcal meningitis

78 Were assigned to receive amphotericin B for 2 weeks

39 Were assigned to receive amphotericin B plus fluconazole

39 Were assigned to receive

amphotericin B plus flucytosine

73 Were eligible for the trial

5 Were excluded

1 Had previous cryptococcal meningitis

3 Met late-exclusion criteria

1 Withdrew consent

236 Were enrolled and underwent randomization

79 Were assigned to receive amphotericin B for 1 week

38 Were assigned to receive

amphotericin B plus fluconazole

41 Were assigned to receive

amphotericin B plus flucytosine

76 Were eligible for the trial

3 Were excluded

1 Did not have confirmed cryptococcal meningitis

2 Met late-exclusion criteria

76 Were included in the intention-to- treat analysis

36 Received amphotericin B plus fluconazole

40 Received amphotericin B plus flucytosine

75 Were included in the per-protocol analysis

74 Were included in the per-protocol analysis

35 Received amphotericin B plus fluconazole

39 Received amphotericin B plus flucytosine

69 Were included in the per-protocol analysis

35 Received amphotericin B plus fluconazole

34 Received amphotericin B plus flucytosine

2 Were excluded

1 Did not receive correct treatment

1 Did not complete assigned treatment

4 Were excluded owing to not completing assigned treatment

# Figure 1. Screening, Randomization, and Analysis Populations

* Patients may have had more than one reason for exclusion. ART denotes antiretroviral therapy, and HIV human immunodeficiency virus.

# Table 1. Baseline characteristics of study participants by 5 treatment arms (N=224)

| **Baseline characteristics** | **Oral**  **(2 weeks 5FC+FLU)**  **(n=75)** | **1 week AmB**  **+ FLU**  **(n=36)** | **1 week AmB**  **+ 5FC**  **(n=40)** | **2 weeks AmB**  **+ FLU**  **(n=36)** | **2 weeks AmB**  **+ 5FC**  **(n=37)** |
| --- | --- | --- | --- | --- | --- |
| **Male sex − no. (%)** | 41 (54.7) | 23 (63.9) | 25 (62.5) | 24 (66.7) | 24 (64.9) |
| **Age − years**  **Median (IQR)** | 37.0 (32.0 to 0.0) | 41.5 (35.5 to 46.5) | 37.0 (30.5 to 42.0) | 37.5 (32.0 to 42.5) | 37.0 (30.0 to 43.0) |
| **Reported ART exposure − no. (%)** | 46 (61.3) | 18 (50.0) | 24 (60.0) | 25 (69.4) | 28 (75.7) |
| **Weight (kg) ^Φ^**  **Median (IQR)** | 50 (45.0 to 55.0) | 55 (49.0 to 61.5) | 52 (45.0 to 60.0) | 50 (46.5 to 56.5) | 50 (46.0 to 60.0) |
| **Current headache − no. (%)** | 74 (98.7) | 36 (100) | 40 (100) | 36 (100) | 37 (100) |
| **Duration of headache (days)^¶^**  **Median (IQR)** | 14 (7.0 to 21.0) | 14 (7.0 to 14.0) | 10 (7.0 to 20.5) | 14 (10.0 to 28.0) | 14 (7.0 to 21.0) |
| **Seizures within 72hrs of enrolment − no. (%)** | 19 (25.3) | 8 (22.2) | 5 (12.5) | 9 (25.0) | 8 (21.6) |
| **Current fever − no. (%)** | 10 (13.3) | 5 (13.9) | 2 (5.0) | 3 (8.3) | 7 (18.9) |
| **Current visual loss − no. (%)** | 3 (4.0) | 2 (5.6) | 1 (2.5) | 3 (8.3) | 4 (10.8) |
| **Cranial to nerve palsy (any) − no. (%)** | 2 (2.7) | 2 (5.6) | 3 (7.5) | 4 (11.1) | 5 (13.5) |
| **Previous medical history of TB –**  **no. (%)** | 15 (20.0) | 7 (19.4) | 12 (30.0) | 10 (27.8) | 10 (24.1) |
| **Glasgow coma score < 15 − no. (%)** | 20 (26.7) | 13 (36.1) | 9 (22.5) | 12 (33.3) | 9 (24.3) |
| **Abnormal mental status − no. (%)** | 29 (38.7) | 14 (38.9) | 12 (30.0) | 19 (52.8) | 18 (48.7) |
| **CSF fungal count (log_10_CFU/ml) ^ξ^** |  |  |  |  |  |
| **Median (IQR)** | 5.0 (3.5 to 5.7) | 5.0 (3.3 to 6.4) | 5.0 (3.6 to 5.9) | 5.1 (4.0 to 5.9) | 5.0 (3.9 to 5.4) |
| **CSF opening pressure (cm)^β^** |  |  |  |  |  |
| **Median (IQR)** | 22.0 (16.0 to 30.0) | 25.0 (14.0 to 40.0) | 25.5 (10.0 to 40.0) | 30.0 (17.0 to 40.0) | 19.5 (15.0 to 30.5) |
| **CSF opening pressure (cm )^β^** |  |  |  |  |  |
| **>30 – no. (%)** | 21 (29.6) | 15 (42.9) | 17 (44.7) | 17 (51.5) | 10 (27.8) |
| **CSF white cell count (cells/ml) ^Ω^** |  |  |  |  |  |
| **Median (IQR)** | 8.0 (0.0 to 64.5) | 6.5 (3.0 to 40.0) | 4.0 (0.0 to 35.0) | 6.0 (0.0 to 43.0) | 4.0 (0.0 to 44.0) |
| **CSF glucose level (mmol/L) ^ϑ^**  **Median (IQR)** | 2.0 (1.0 to 2.0) | 2.0 (1.0 to 3.0) | 2.0 (1.0 to 2.0) | 2.0 (1.0 to 2.0) | 1.2 (1.0 to 2.0) |
| **CSF protein level (mg/dL)^**^**  **Median (IQR)** | 107 (46.0 to 218.0) | 98 (58.0 to 148.0) | 89 (53.0 to 177.0) | 103.5 (52.5 to 147.0) | 86 (54.0 to 215.0) |
| **Hemoglobin (g/dl)^♯^**  **Median (IQR)** | 11.1 (9.3 to 12.9) | 11.4 (10.4 to 12.8) | 12.0 (10.7 to 13.1) | 11.9 (10.6 to 13.2) | 11.2 (9.9 to 12.7) |
| **Creatinine (umol/L) ^Ξ^**  **Median (IQR)** | 61.6 (52.8 to 70.4) | 70.7 (61.7 to 83.1) | 61.6 (48.1 to 77.4) | 61.9 (52.9 to 83.8) | 61.8 (53.0 to 82.3) |
| **Baseline CD4 count (cells/uL) ^ς^**  **Median (IQR)** | 20.0 (9.0 to 63.5) | 29.0 (16.0 to 74.0) | 26.0 (9.0 to 64.0) | 16.0 (5.0 to 57.0) | 32.5 (10.5 to 73.0) |

**^Φ^** Data were missing for 1 patient in 1 week AmB+5FC.

**^¶^** Data were missing for 1 patient in oral.

**^ξ^** Data were missing for 5 patients in oral, 2 patients in 1 week AmB+5FC, 4 patients in 2 weeks AmB+FLU and 1 patient in 2 weeks AmB+5FC.

**^β^** Data were missing for 4 patients in oral, 1 patients in 1 week AmB+FLU, 2 patients in 1 week AmB+5FC, 3 patients in 2 weeks AmB+FLU and 1 patient in 2 weeks AmB+5FC.

**^Ω^** Data were missing for 3 patients in oral, 2 patients in 1 week AmB+FLU, 3 patients in 1 week AmB+5FC, 2 patients in 2 weeks AmB+FLU and 3 patients in 2 weeks AmB+5FC.

**^ϑ^** Data were missing for 7 patients in oral, 2 patients in 1 week AmB+5FC, 4 patients in 2 weeks AmB+FLU and 3 patients in 2 weeks AmB+5FC.

**^**^** Data were missing for 3 patients in oral, 2 patients in 1 week AmB+5FC, 4 patients in 2 weeks AmB+FLU and 3 patients in 2 weeks AmB+5FC.

**^♯^** Data were missing for 3 patients in oral and 1 patient in 2 weeks AmB+5FC.

**^Ξ^** Data were missing for 3 patients in oral and 1 patient in 2 weeks AmB+5FC.

**^ς^** Data were missing for 3 patients in oral, 1 patient in 2 weeks AmB+FLU and 1 patients in 2 weeks AmB+5FC.

#

|  |  |  |  |  |  | **Hazard Ratio (95% CI) (Reference group: 1 week AmB + 5FC)** | | | | |
| --- | --- | --- | --- | --- | --- | --- | --- | --- | --- | --- |
| **Outcomes** | **Oral**  **(2 weeks 5FC+FLU)**  **(n=75)** | **1 week AmB**  **+ FLU**  **(n=36)** | **1 week AmB**  **+ 5FC**  **(n=40)** | **2 weeks AmB**  **+ FLU**  **(n=36)** | **2 weeks AmB**  **+ 5FC**  **(n=37)** | **Oral**  **(2 weeks 5FC+FLU)** | **1 week AmB**  **+ FLU** | **2 weeks AmB**  **+ FLU** | **2 weeks AmB**  **+ 5FC** | **p-value**  **(log-rank test)** |
| **1 year mortality** |  |  |  |  |  |  |  |  |  |  |
| No. of deaths  Probability of death  (95% CI) | 33  44.0  (33.7 to 55.9) | 22  61.1  (45.8 to 76.7) | 11  27.5  (16.3 to 44.1) | 18  50.0  (35.1 to 67.1) | 17  46.0  (31.6 to 63.1) | 1.57  (0.79 to 3.11) | 2.73  (1.32 to 5.67) | 1.99  (0.93 to 4.24) | 1.90  (0.88 to 4.08) | 0.04 |
| **6 month mortality** |  |  |  |  |  |  |  |  |  |  |
| No. of deaths  Probability of death  (95% CI) | 27  36.0  (26.3 to 47.9) | 20  55.6  (40.4 to 72.0) | 11  27.5  (16.3 to 44.1) | 17  47.2  (32.6 to 64.5) | 17  46.0  (31.6 to 63.1) | 1.26  (0.62 to 2.55) | 2.40  (1.14 to 5.06) | 1.79  (0.83 to 3.85) | 1.84  (0.86 to 3.95) | 0.06 |
| **10 week mortality** |  |  |  |  |  |  |  |  |  |  |
| No. of deaths  Probability of death  (95% CI) | 23  30.7  (21.6 to 42.4) | 19  52.8  (37.7 to 69.5) | 9  22.5  (12.4 to 38.8) | 14  38.9  (25.2 to 56.7) | 15  40.5  (26.7 to 58.0) | 1.31  (0.60 to 2.84) | 2.70  (1.21 to 6.04) | 1.75  (0.75 to 4.08) | 1.91  (0.83 to 4.39) | 0.04 |

# Table 2. Time to event outcomes by 5 treatment arms (Intention-to-treat, adjusted analysis, 1 week AmB+5FC as reference; N=224)

# Table 3. Baseline characteristics of study participants by 3 treatment regimens (N=224)

| **Baseline characteristics** | **Oral**  **(2 weeks 5FC+FLU)**  **(n=75)** | **1 week AmB**  **(n=76)** | **2 weeks AmB**  **(n=73)** |
| --- | --- | --- | --- |
| **Male sex − no. (%)** | 41 (54.7) | 48 (63.2) | 48 (65.8) |
| **Age − years**  **Median (IQR)** | 37.0 (32.0 to 40.0) | 38.5 (32.0 to 44.5) | 37.0 (31.0 to 43.0) |
| **Reported ART exposure − no. (%)** | 46 (61.3) | 42 (55.3) | 53 (72.6) |
| **Weight (kg) ^Φ^**  **Median (IQR)** | 50.0 (45.0 to 55.0) | 55.0 (46.0 to 60.0) | 50.0 (46.0 to 60.0) |
| **Current headache − no. (%)** | 74 (98.7) | 76 (100.0) | 73 (100.0) |
| **Duration of headache (days)^¶^**  **Median (IQR)** | 14 (7 to 21) | 12 (7 to 24) | 14 (7 to 24) |
| **Seizures within 72hrs of enrolment − no. (%)** | 19 (25.3) | 13 (17.1) | 17 (23.3) |
| **Current fever − no. (%)** | 10 (13.3) | 7 (9.21) | 10 (13.7) |
| **Current visual loss − no. (%)** | 3 (4.0) | 3 (4.0) | 7 (9.6) |
| **Cranial to nerve palsy (any) − no. (%)** | 2 (2.7) | 5 (6.6) | 9 (12.3) |
| **Previous medical history of TB –**  **no. (%)** | 15 (20.0) | 19 (25.0) | 20 (27.4) |
| **Glasgow coma score < 15 − no. (%)** | 20 (26.7) | 22 (29.0) | 21 (28.8) |
| **Abnormal mental status − no. (%)** | 29 (38.7) | 26 (34.2) | 37 (50.7) |
| **CSF fungal count (log_10_CFU/ml) ^ξ^** |  |  |  |
| **Median (IQR)** | 5.0 (3.5 to 5.7) | 5.0 (3.5 to 6.0) | 5.0 (3.8 to 5.7) |
| **CSF opening pressure (cm )^β^** |  |  |  |
| **Median (IQR)** | 22 (16 to 30) | 25 (12 to 40) | 22 (15 to 38) |
| **CSF opening pressure* (cm )^β^** |  |  |  |
| **>30- no. (%)** | 21 (29.6) | 32 (43.8) | 27 (39.1) |
| **CSF white cell count (cells/ml) ^Ω^** |  |  |  |
| **Median (IQR)** | 8.0 (0.0 to 64.5) | 5.0 (0.0 to 40.0) | 4.0 (0.0 to 43.5) |
| **CSF glucose level (mmol/L)^ϑ^**  **Median (IQR)** | 2.0 (1.0 to 2.0) | 2.0 (1.0 to 2.6) | 1.7 (1.0 to 2.0) |
| **CSF protein level (mg/dL)^**^**  **Median (IQR)** | 107 (46 to 218) | 96 (55 to 157) | 99 (54 to 169) |
| **Hemoglobin (g/dl)^♯^**  **Median (IQR)** | 11.1 (9.3 to 12.9) | 11.7 (10.6 to 13.0) | 11.5 (10.1 to 13.0) |
| **Creatinine (umol/L)^Ξ^**  **Median (IQR)** | 61.6 (52.8 to 70.4) | 66.1 (53.0 to 79.4) | 61.8 (53.0 to 82.3) |
| **Baseline CD4 count (cells/uL)^ς^**  **Median (IQR)** | 20 (9.0 to 63.5) | 28 (12.0 to 69.5) | 24 (6.0 to 63.0) |

**^Φ^** Data were missing for 1 patient in 1 week AmB.

**^¶^** Data were missing for 1 patient in oral.

**^ξ^** Data were missing for 5 patients in oral, 2 patients in 1 week AmB, 5 patients in 2 weeks AmB.

**^β^** Data were missing for 4 patients in oral, 3 patients in 1 week AmB and 4 patients in 2 week AmB.

**^Ω^** Data were missing for 3 patients in oral, 5 patients in 1 week AmB and 5 patients in 2 week AmB.

**^ϑ^** Data were missing for 7 patients in oral, 2 patients in 1 week AmB and 7 patients in 2 week AmB.

**^**^** Data were missing for 3 patients in oral, 2 patients in 1 week AmB and 7 patients in 2 week AmB.

**^♯^** Data were missing for 3 patients in oral and 1 patient in 2 weeks AmB.

**^Ξ^** Data were missing for 3 patients in oral and 1 patient in 2 weeks AmB.

**^ς^** Data were missing for 3 patients in oral and 2 patients in 2 weeks AmB.

# Table 4. Time to event outcomes by 3 treatment regimens (Intention-to-treat, unadjusted analysis; N=224)

|  | |  | |  | |  | **Hazard Ratio (95% CI) (Reference group: 2 weeks AmB)** | | |
| --- | --- | --- | --- | --- | --- | --- | --- | --- | --- |
| **Outcomes** | **Oral (2 weeks 5FC+FLU)**  **(n=75)** | | **1 week AmB**  **(n=76)** | | **2 weeks AmB**  **(n=73)** | | **Oral**  **(2 weeks 5FC+FLU)** | **1 week AmB**  **+ FLU** | **p-value**  **(log-rank test)** |
| **1 year mortality** |  | |  | |  | |  |  |  |
| No. of deaths  Probability of death (95% CI) | 33  44.0  (32.6 to 55.9) | | 33  43.4  (32.1 to 55.3) | | 35  47.9  (36.1 to 60.0) | | 0.85  (0.53 to 1.36) | 0.91  (0.57 to 1.47) | 0.79 |
| **6 month mortality** |  | |  | |  | |  |  |  |
| No. of deaths  Probability of death (95% CI) | 27  36.0  (25.2 to 47.9) | | 31  40.8  (29.6 to 52.7) | | 34  46.6  (34.8 to 58.6) | | 0.72  (0.43 to 1.45) | 0.89  (0.55 to 1.45) | 0.43 |
| **10 week mortality** |  | |  | |  | |  |  |  |
| No. of deaths  Probability of death (95% CI) | 23  30.7  (21.5 to 42.4) | | 28  36.8  (26.1 to 48.7) | | 29  39.7  (28.5 to 51.9) | | 0.73  (0.42 to 1.26) | 0.96  (0.57 to 1.61) | 0.48 |

# Table 5. Baseline characteristics of study participants by AmB partner treatment (N=149)

| **Baseline characteristics** | **AmB + FLU**  **(n=72)** | **AmB + 5FC**  **(n=77)** |
| --- | --- | --- |
| **Male sex − no. (%)** | 47 (65.3) | 49 (63.4) |
| **Age − years**  **Median (IQR)** | 39.5 (32.5 to 45.0) | 37.0 (30.0 to 42.0) |
| **Reported ART exposure − no. (%)** | 43 (59.7) | 52 (67.5) |
| **Weight (kg)^Φ^**  **Median (IQR)** | 53.5 (47.0 to 60.0) | 50.5 (45.0 to 60.0) |
| **Current headache − no. (%)** | 72 (100.0) | 77 (98.7) |
| **Duration of headache (days)**  **Median (IQR)** | 14.0 (7.0 to 20.5) | 12.0 (7.0 to 21.0) |
| **Seizures within 72hrs of enrolment − no. (%)** | 17 (23.6) | 13 (16.9) |
| **Current fever − no. (%)** | 8 (11.1) | 11 (11.7) |
| **Current visual loss − no. (%)** | 5 (6.9) | 5 (6.5) |
| **Cranial-nerve palsy (any) − no. (%)** | 6 (8.3) | 8 (10.4) |
| **Previous medical history of TB –**  **no. (%)** | 17 (23.6) | 22 (28.6) |
| **Glasgow coma score < 15 − no. (%)** | 25 (34.7) | 18 (23.4) |
| **Abnormal mental status − no. (%)** | 33 (45.8) | 30 (39.0) |
| **CSF fungal count (log_10_CFU/ml) ^ξ^** |  |  |
| **Median (IQR)** | 5.1 (3.7 to 5.9) | 5.0 (3.8 to 5.8) |
| **CSF opening pressure (cm ) ^β^** |  |  |
| **Median (IQR)** | 26.0 (15.0 to 40.0) | 20.0 (12.0 to 39.0) |
| **CSF opening pressure (cm ) ^β^** |  |  |
| **>30 - no. (%)** | 32 (47.1) | 27 (36.5) |
| **CSF white cell count (cells/ml)^Ω^** |  |  |
| **Median (IQR)** | 6.5 (1.5 to 41.5) | 4.0 (0.0 to 44.0) |
| **CSF glucose level (mmol/L)^ϑ^**  **Median (IQR)** | 2.0 (1.0 to 2.5) | 2.0 (1.0 to 2.0) |
| **CSF protein level (mg/dL)^**^**  **Median (IQR)** | 99.0 (55.0 to 147.0) | 89.0 (53.5 to 193.5) |
| **Hemoglobin (g/dl)^♯^**  **Median (IQR)** | 11.8 (10.4 to 13.1) | 11.5 (10.4 to 13.0) |
| **Creatinine (umol/L)^Ξ^**  **Median (IQR)** | 70.4 (53.0 to 83.1) | 61.8 (52.9 to 79.2) |
| **Baseline CD4 count (cells/uL)^ς^**  **Median (IQR)** | 25.0 (6.0 to 62.0) | 29.0 (9.5 to 67.5) |

**^Φ^** Data were missing for 1 patient in AmB+5FC

**^ξ^** Data were missing for 4 patients in AmB+FLU and 3 patients in AmB+5FC

**^β^** Data were missing for 4 patients in AmB+FLU and 3 patients in AmB+5FC.

**^β^** Data were missing for 4 patients in AmB+FLU and 3 patients in AmB+5FC.

**^Ω^** Data were missing for 4 patients in AmB+FLU and 6 patients in AmB+5FC.

**^ϑ^** Data were missing for 4 patients in AmB+FLU and 5 patients in AmB+5FC.

**^**^** Data were missing for 4 patients in AmB+FLU and 5 patients in AmB+5FC.

**^♯^** Data were missing for 1 patient in AmB+5FC

**^Ξ^** Data were missing for 1 patient in AmB+5FC

**^ς^** Data were missing for 1 patients in AmB+FLU and 1 patient in AmB+5FC.

# Table 6. Time to event outcomes by AmB partner treatment (Intention-to-treat, unadjusted analysis; N=149)

|  | | | **Hazard Ratio (95% CI)** | | |
| --- | --- | --- | --- | --- | --- |
| **Outcome** | **AmB + FLU**  **(n=72)** | **AmB + 5FC**  **(n=77)** | | **AmB + 5FC**  **vs**  **AmB + FLU** | **p-value**  **(log-rank test)** |
| **1 year mortality** |  |  | |  |  |
| No. of deaths  Probability of death  (95% CI) | 40  55.6  (44.6 to 67.2) | 28  36.4  (26.7 to 48.1) | | 0.56  (0.34 to 0.91) | 0.02 |
| **6 month mortality** |  |  | |  |  |
| No. of deaths  Probability of death  (95% CI) | 37  51.4  (40.5 to 63.3) | 28  36.4  (26.7 to 48.1) | | 0.61  (0.37 to 1.00) | 0.06 |
| **10 week mortality** |  |  | |  |  |
| No. of deaths  Probability of death  (95% CI) | 33  45.8  (35.2 to 58.0) | 24  31.2  (22.1 to 42.8) | | 0.59  (0.35 to 1.00) | 0.05 |


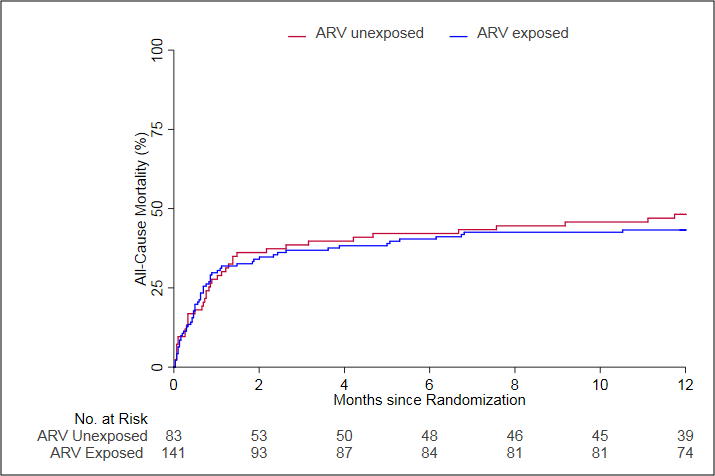


# Figure 2. All-Cause Mortality by ARV Exposure

Figure 2 shows cumulative all-cause mortality by ARV exposure up to 1 year post randomization (log rank p-value: 0.58).
